# Characterization and Metabolic Diversity of Flavonoids in Citrus Species

**DOI:** 10.1038/s41598-017-10970-2

**Published:** 2017-09-05

**Authors:** Shouchuang Wang, Chenkun Yang, Hong Tu, Junjie Zhou, Xianqing Liu, Yunjiang Cheng, Jie Luo, Xiuxin Deng, Hongyan Zhang, Juan Xu

**Affiliations:** 1 National Key Laboratory of Crop Genetic Improvement and National Center of Plant Gene Research (Wuhan), Huazhong Agricultural University, Wuhan, 430070 China; 2 Key Laboratory of Horticultural Plant Biology (Ministry of Education), College of Horticulture and Forestry Sciences, Huazhong Agricultural University, Wuhan, 430070 China; 3 College of Life Science and Technology, Huazhong Agricultural University, Wuhan, 430070 China

## Abstract

Flavonoids are widely distributed in plants and play important roles in many biological processes. Citrus fruits are rich dietary sources of flavonoids. However, there have been very few reports about the comprehensive metabolic profile and natural diversity of flavonoids in different tissues of various *Citrus* cultivars. In this study, based on the 7416 metabolic signals detected with non-targeted metabolomics approach, Principal Component Analysis revealed the flavedo has the largest differences from other tissues in metabolite levels; as many as 198 flavonoid signals were then detected in 62 *Citrus* germplasms from 5 species mainly cultivated worldwide, while 117 flavonoids were identified, including 39 polymethoxylated flavonoids (PMFs), 7 flavones, 10 *C*-*O*-glycosylflavonoids, 44 *O*-glycosylflavonoids, 10 *C*-glycosylflavonoids and 7 newly annotated *O*-glycosylpolymethoxylated flavonoids. Tissue-specific accumulations were observed: *O*-glycosylated flavonoids were abundant in all fruit tissues, while PMFs were accumulated preferentially in the flavedo. Among different species, mandarins had the highest levels of PMFs and *O*-glycosylpolymethoxylated flavonoids, followed by sweet oranges. Based on the flavonoid profiles, 62 germplasms could be clearly grouped into five distinct clusters via hierarchical clustering analysis, which were perfectly matched with their species, with sweet oranges and mandarins clustering closely and being further away from other three species.

## Introduction

Flavonoids, a group of important secondary metabolites, are widely distributed in the plant kingdom, and as many as 6000 flavonoid-related compounds have been identified^1, 2^. In plants, flavonoids along with other phenylpropanoids are synthesized from phenylalanine, including the subgroups of flavanones, flavones, isoflavones, flavanols, flavonols and anthocyanidins^3–5^. It has been reported that flavonoids play important roles in some physiological processes^6, 7^ and exhibit a wide range of promising pharmaceutical properties for human health, including anti-atherogenic, anti-inflammatory, antitumor and antioxidant activities and inhibitory activity against blood clots^8, 9^. Currently, the main source of flavonoids for human beings is still dietary intake, and flavonoids are present in most edible fruits, vegetables^10^ and cereals^6^.

As one of the most widely cultivated fruit crops in the world, citrus fruits are rich in flavonoids, vitamin C, folate, dietary fiber and carotenoids^5^. The most widely cultivated *Citrus* are mandarins, oranges, pummelos, grapefruits and lemons^11^. As one of the most important origin centers, China has many native wild citrus species and important varieties^12–14^, such as some pummelos and wild mandarins^15, 16^. Citrus fruits are one of the most important dietary sources of flavonoids, especially poly-methoxyflavones (PMFs)^17^. However, the genetic mechanisms governing the synthesis, modification and distribution of flavonoids remain largely unknown^18^.

Flavonoids are present in various modified forms corresponding to additional hydroxylation^19^, methylation^17^ and, most importantly, glycosylation in *Citrus* ^1^. The major flavonoids in citrus fruits are flavanone-*O*-glycosides, flavone-*O*/*C*-glycosides and their derivatives^11, 20^. Flavanones are very important citrus flavonoids, and some are responsible for citrus bitterness, such as naringin, neohesperidin, neoeriocitrin and poncirin, which have significant impacts on the sensory quality of citrus fruits^21^. Many studies have shown that PMFs and flavone *O*-glycosides are the most common flavones in citrus fruit, and their aglycones are apigenin, diosmetin and luteolin^11^. A small number of *C*-glycosyl flavones have also been detected in blood orange^22^. PMFs, one special group of flavonoids in which almost all hydroxyls are capped by methylation, are predominantly present in *Citrus* genus, especially in the peels of sweet oranges and wild mandarins^23, 24^. PMFs glycosides were identified in *Murraya paniculata* leaves, which was the first time that the presence of PMFs glycosides in the genus was reported^25^. There have been no related reports about the glycosylation of PMFs in *Citrus* genus so far.

Owing to the varied flavonoid compositions among different citrus species^20, 26^ and tissues^10, 27^, flavonoids can be taken as a metabolic marker to distinguish citrus varieties, and can be applied to fruit juice identification. For example, the content of hesperetin is a marker of the floral origin of citrus honey^28^, while for lemon juice, flavonoids such as eriodictyol-7-*O*-rutinoside, diosmetin-6, 8-di-*C*-glucoside, diosmetin-8-*C*-glucoside, luteolin-7-*O*-rutinoside and diosmetin-6-*C*-glucoside are taken as marker metabolites^20^.

As a powerful method, HPLC–MS–based widely targeted metabolomics has been successfully used for specific detection of flavonoids and characterization of new flavonoids in *Arabidopsis* and rice^6, 29, 30^. In a previous study, eight new flavonoids were identified by metabolic profiling of flavonoids in bergamot juice^26^. The phenolic compounds in citrus juices from Spanish cultivars were also comprehensively characterized with the same method^31^. Despite this progress, the flavonoid metabolism is not systematically studied, and their naturally occurring variation in citrus germplasm remains elusive.

In our research, the different accumulation patterns of flavonoids and its metabolic diversity were carried out in 62 *Citrus* germplasms from five most widely cultivated *Citrus* species, including one wild germplasm. Unlike previous studies, the aim of this study was to determine the content and composition of flavonoid compounds in citrus fruits, provide comprehensive profiling of flavonoids in different citrus species, and reveal some metabolic diversities of flavonoids in citrus fruits. We have only focused on 117 flavonoids contained in citrus, then provided more detailed data in various citrus germplasms; this work will not only benefit the evaluation of existed germplasms, but also shed light on future selection and breeding of new healthy citrus germplasms. Although 36 compounds have been previously described^32^, 7 new compounds were detected for the first time in citrus in this work. 117 flavonoids were (tentatively) identified and the tissue-specific accumulations were detected for most flavonoids. The neighbor-joining tree based on flavonoid profiles has been used to study the population structure of citrus from the metabolic level. The results pave the way for future dissection of biosynthesis and genetic regulation of flavonoid metabolic pathways in citrus, and could be helpful in the selection of breeding parents for new specific flavonoid-rich germplasms.

## Results and Discussion

### Metabolic profiles of Citrus fruits

For comprehensive profiling of metabolites in citrus fruits, we analyzed 62 *Citrus* germplasms representing five major species in *Citrus* genus, including sweet oranges (SO), mandarins (M), lemons (L), pummelos (P) and grapefruits (G) (Supplementary Table S1). Non-targeted high-performance liquid chromatography with diode array detection and electrospray ionization mass spectrometry (HPLC-DAD-ESI-MS/MS) was used to profile the metabolites in fruit tissues. As a result, 7416 metabolic signals were detected (Supplementary Table S2).

**Figure 1:**
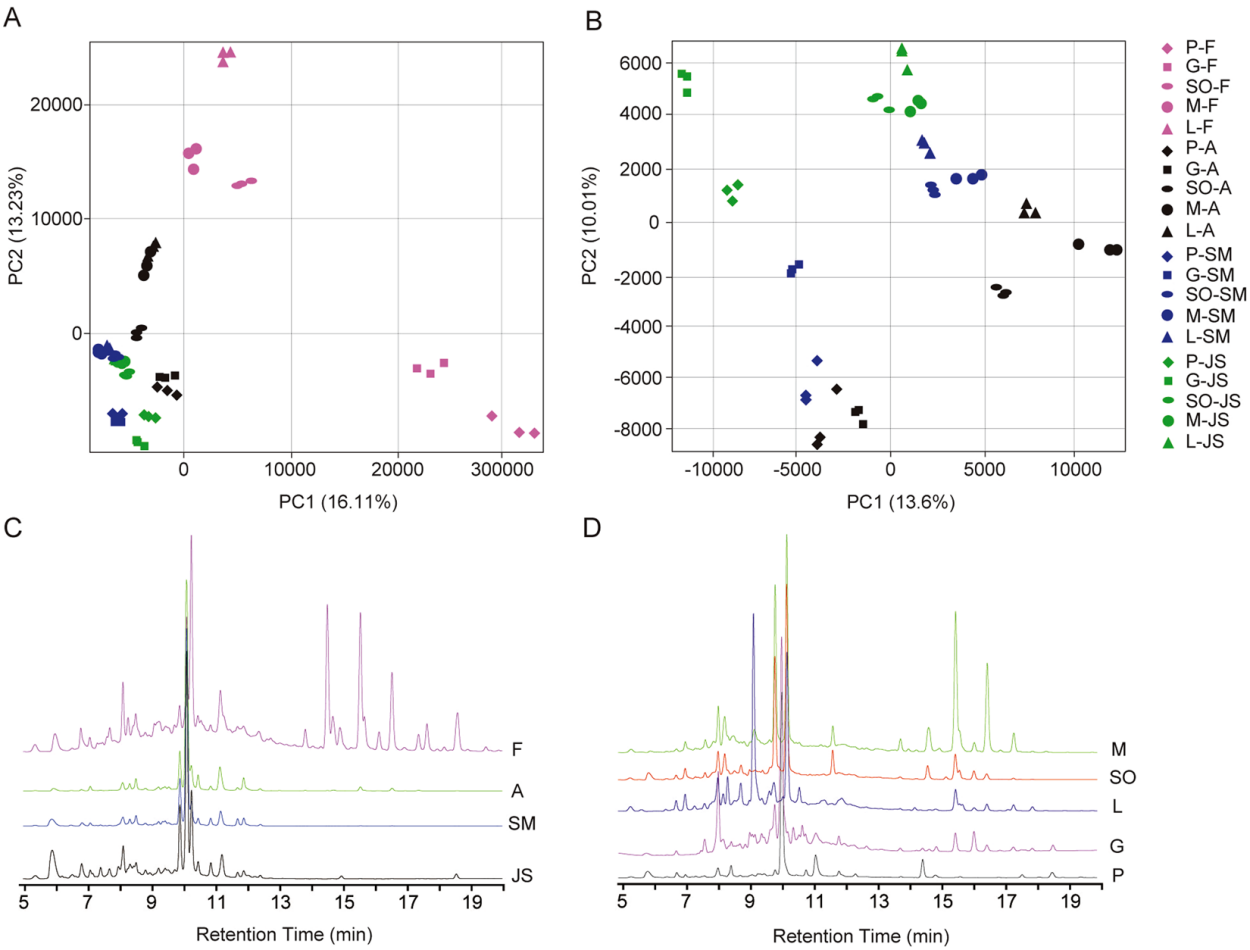
Analysis of metabolic differences in different tissues and varieties using HPLC–DAD-QqTOF–MS/MS. (**A**) PCA results of the metabolites in four citrus tissues. (**B**) PCA results of the metabolites in albedo, SM and JS. (**C**) HPLC–DAD chromatograms of metabolites in flavedo, albedo, SM and JS at 280 nm. (**D**) HPLC–DAD chromatograms of metabolites in five species at 280 nm. The level of significance was set at *P* < 0.01. The samples of the four tissues of flavedo, albedo, SM and JS were displayed with pink, black, blue and green symbols, respectively.

To investigate the inter- and intra-species metabolic differences, mixed samples of different fruit tissues, including the flavedo (F), albedo (A), segment membrane (SM) and juice sacs (JS) from five *Citrus* species, were prepared and subjected to metabolic profiling via Liquid Chromatography Tandem Time of Flight Mass Spectrometer (LC-TOF-MS). Based on the untargeted metabolomics and Mass Profiler Professional (MPP) analysis, Principal Component Analysis (PCA), an unsupervised method, was subsequently used to briefly evaluate the kinetic metabolome patterns of different fruit tissues and *Citrus* species. PCA clearly grouped these tissues into four distinct clusters in individual *Citrus* species, while classified the metabolic signals from the same tissues of different species into five distinct clusters (Fig. 1A and B). The first three main PCs (PC1, PC2 and PC3) explained the variability of the entire system by 33.08–39.13%, and all variables contributed to the PCs were showed in Supplementary Table S2.

Furthermore, PCA separated the flavedo from other three tissues in all the five species, indicating that the flavedo has the largest differences from other tissues in metabolite levels (Fig. 1A). Notably, all the four tissues of various species showed similar patterns in their metabolomes. For the outermost tissue flavedo, the plots were widely scattered, suggesting that metabolomes considerably vary among different species (Fig. 1A). However, for the innermost tissues SM and JS, the plots were much less distinguishable, indicating similar metabolic profiles between the two tissues (Fig. 1B). Furthermore, the PCA plots of the same tissue from the five species were relatively closer, indicating lower metabolic variations within the same tissues of different species.

### Flavonoid identification using HPLC-DAD-ESI-MS/MS

To further identify flavonoids, multiple Diode Array Detector (DAD) wavelength-scanning programs were used, which were capable of monitoring several wavelengths simultaneously. The flavonoid signals for total ions at 280 nm were listed in Fig. 1C and D. Based on the UV absorbance and mass spectrometric data, in addition to the publically available information of flavonoids, 198 precursor ions were obtained to construct the flavonoid metabolism database in this study (Supplementary Table S3).

In order to better identify the detected flavonoids, 198 flavonoid-related candidate ions were analyzed by using the targeted MS^2^ mode, and the corresponding fragmentation patterns were obtained. Subsequently, a MS^2^ spectral tag (MS2T) library containing 198 metabolite signals of flavonoids with the product ion spectra (MS^2^) was created, which was then annotated based on the fragmentation pattern, retention time (RT), and accurate *m/z* value (Supplementary Table S3).

**Figure 2:**
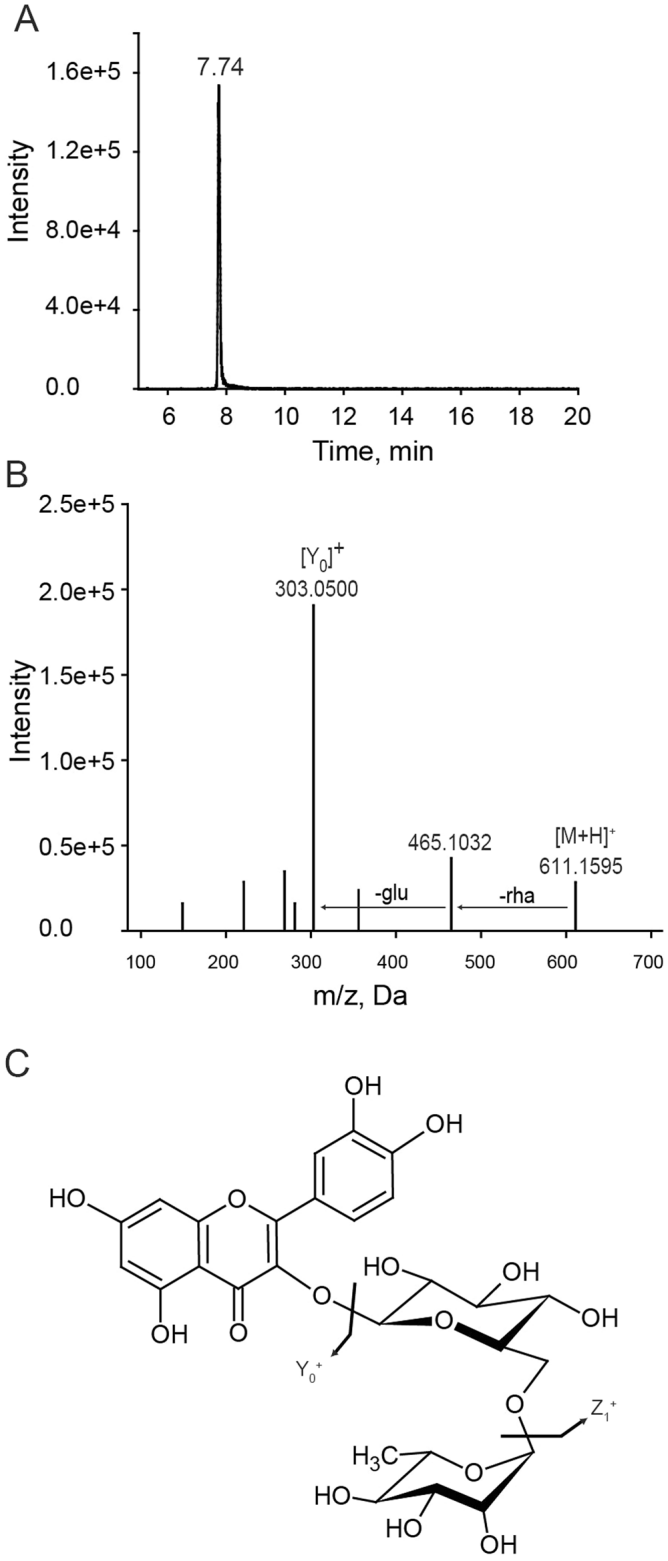
Characterization of flavonoid metabolites in the citrus by HPLC-Q-TOF-MS/MS. (**A**) XIC (extracted ion chromatogram) of compound m149. (**B**) The mass spectrometry information of m/z 611.1595 obtained by the targeted MS^2^ mode, and characterized as quercetin 3-*O*-rutinoside by comparison of the standard. (**C**) The molecular structure of the quercetin 3-*O*-rutinoside and its general fragmentation rules.

Flavonoids with available commercial standards were identified by direct comparison of the *m/z* values, RT, and the secondary mass spectral fragment information with those of the standard compounds. For example, firstly, a metabolite signal was detected at RT 7.74 min (compound m149), and the characteristic spectrum suggested that this metabolite was *O*-diglycosylated flavonoid (Fig. 2A); secondly, the accurate *m/z* value (Q1) detected by ESI-QqTOF-MS/MS in positive ionization mode was 611.1595 (base peak); finally, the product ion mass spectrometry of [M + H]^+^ showed same fragment pattern as quercetin 3-*O*-rutinoside compared with the standards, and a peak was observed at m/z 303.0500 because of the loss of sugar moiety on the aglycone (−308, rutinose) (Fig. 2B), as shown by the structure and main fragmentation pathways of the flavonoids (Fig. 2C).

Flavonoids with no available authentic standards were annotated by comparing the MS fragments with those in literatures or databases (MassBank and METLIN). If the peak had a fragmentation pattern similar to what has been published, the best matches were then searched in the Dictionary of Natural Products (DNP) and Kyoto Encyclopedia of Genes and Genomes (KEGG) for possible structures. About 90 metabolites were putatively annotated following the strategy, and most of them were (*C*- or *O*-) glycosylflavonoids and PMFs (Supplemental Table 4).

**Figure 3:**
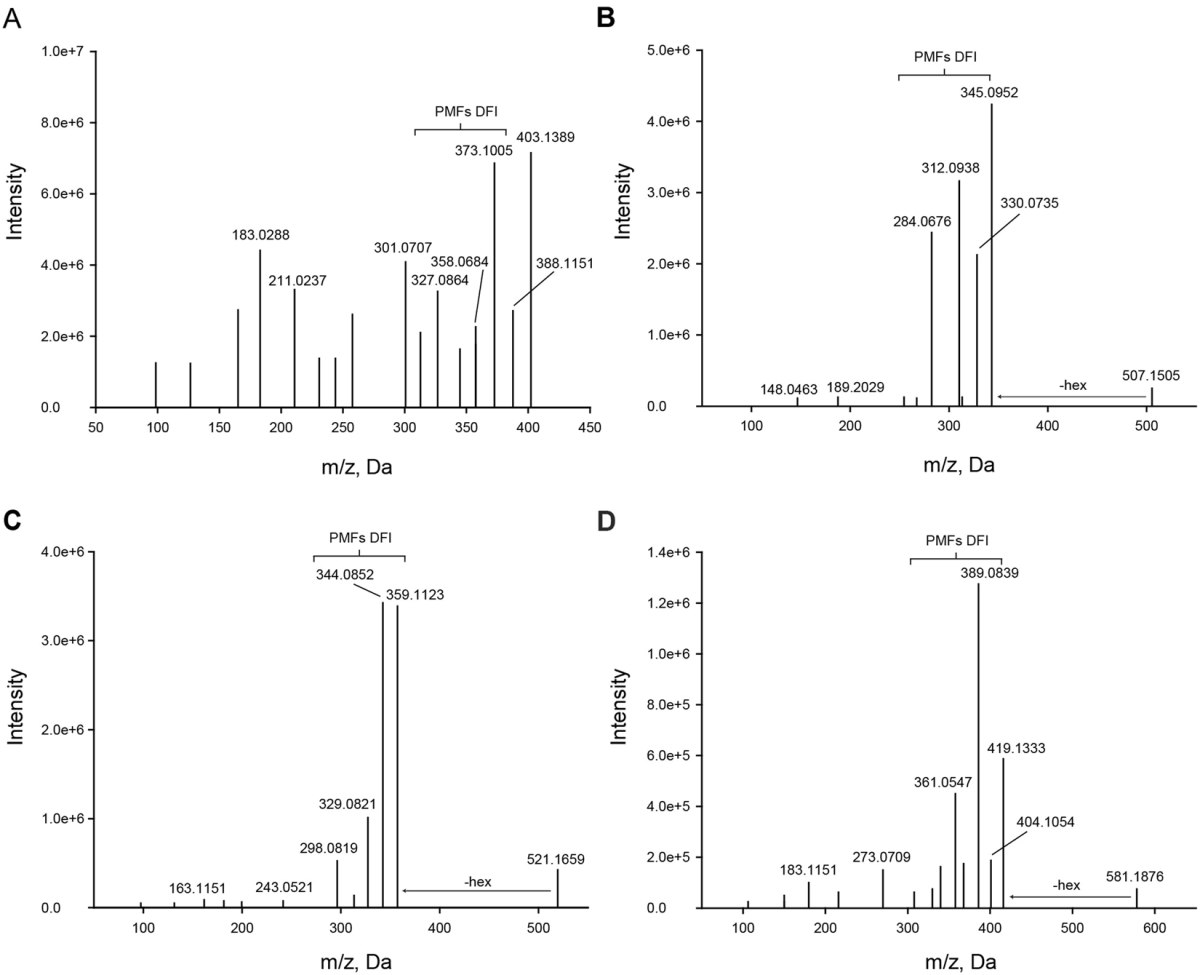
Mass spectra and structures of polymethoxylated flavonoids glycosides in *Citrus*. (**A**) 3′,4′,5,6, 7,8-hexamethoxyflavone (m070). (**B**) Dihydroxy-trimethoxyflavone -*O*-hexoside (m117). (**C**) Hydroxy-tetramethoxyflavone-*O*-hexoside (m119). (**D**) Monohydroxy-hexamethoxyflavone-*O*-hexoside (m133). PMFs DFI, diagnostic fragment ions of polymethoxylated flavonoids.

In addition to those reported metabolites, seven *O*-glycosylpolymethoxylated flavonoids were newly annotated in the study. To better characterize these flavonoids, the 3′,4′,5,6,7,8-hexamethoxyflavone standard (m70, RT 15.3 min, *m/z* 403.1389, error −0.5 ppm) was analyzed first. The precursor ions of the standard compound lost one to four methyl radicals in the MS/MS spectrum to form the base peaks of [M + H − 15]^+^, [M + H − 30]^+^, [M + H − 45]^+^ or [M + H − 60]^+^ (Fig. 3A). Through comparing the product-ion spectra of the standards, some characterized dissociation pathways were found to be involved in the synthesis of the derivatives of both PMFs and *O*-glycosylPMFs. For instance, three *O*-glycosylpolymethoxylated flavonoids newly detected in this study (m117, RT 10.4 min, *m/z* 507.1505, error −1.6 ppm; m119, RT 9.4 min, *m/z* 521.1659, error −1.0 ppm; and m133, RT 12.1 min, *m/z* 581.1876, error −1.9 ppm) were putatively identified as tricetin 5,3′,4′-trimethyl 7-*O*-hexoside, hydroxy-tetramethoxyflavone-*O*-hexoside and monohydroxy-hexamethoxyflavone-*O*- hexoside, respectively. The characteristic loss of 162 Da observed in the MS/MS spectra corresponded to the dissociation of a hexose moiety and a series of methyl loss of the diagnostic fragments of 15 and 30 Da (Fig. 3B–D)^25^. Eventually, seven glycosylated PMFs were detected and annotated in citrus (Supplementary Fig. 1).

Finally, 117 flavonoids were identified /annotated and clearly grouped into 6 main clusters, including 39 polymethoxylated flavonoids (PMFs), 7 *O*-glycosylpolymethoxylated flavonoids (PMFs *O*-gly), 10 *C*-*O*-glycosylflavonoids (Fla *C*, *O*-gly), 44 *O*-glycosylflavonoids (Fla *O*-gly), 7 flavones (Fla), and 10 *C*-glycosylflavonoids (Fla *C*-gly) (Supplementary Table S3). The detected flavonoids were then quantitatively analyzed by multiple reaction monitoring (MRM) under positive mode. (Supplementary Table S4).

### Flavonoid profiles of *Citrus* fruits

At both tissue and specie levels, significant differences in both flavonoid components and contents were found, indicating very large variations of flavonoid metabolism among different tissues and species. Among the four tissues detected, flavonoids were the most abundant in the flavedo tissue, followed by albedo and SM, and the total amount of flavonoids in the JS was the least. Furthermore, consistent with previous reports^10, 18^, the richest flavonoids were found in the flavedo (Fig. 1C). At the species level, the largest number of flavonoid signals was found in the samples of sweet oranges, while the smallest number of flavonoid signals was detected in pummelos and grapefruits (Fig. 1D).

**Figure 4:**
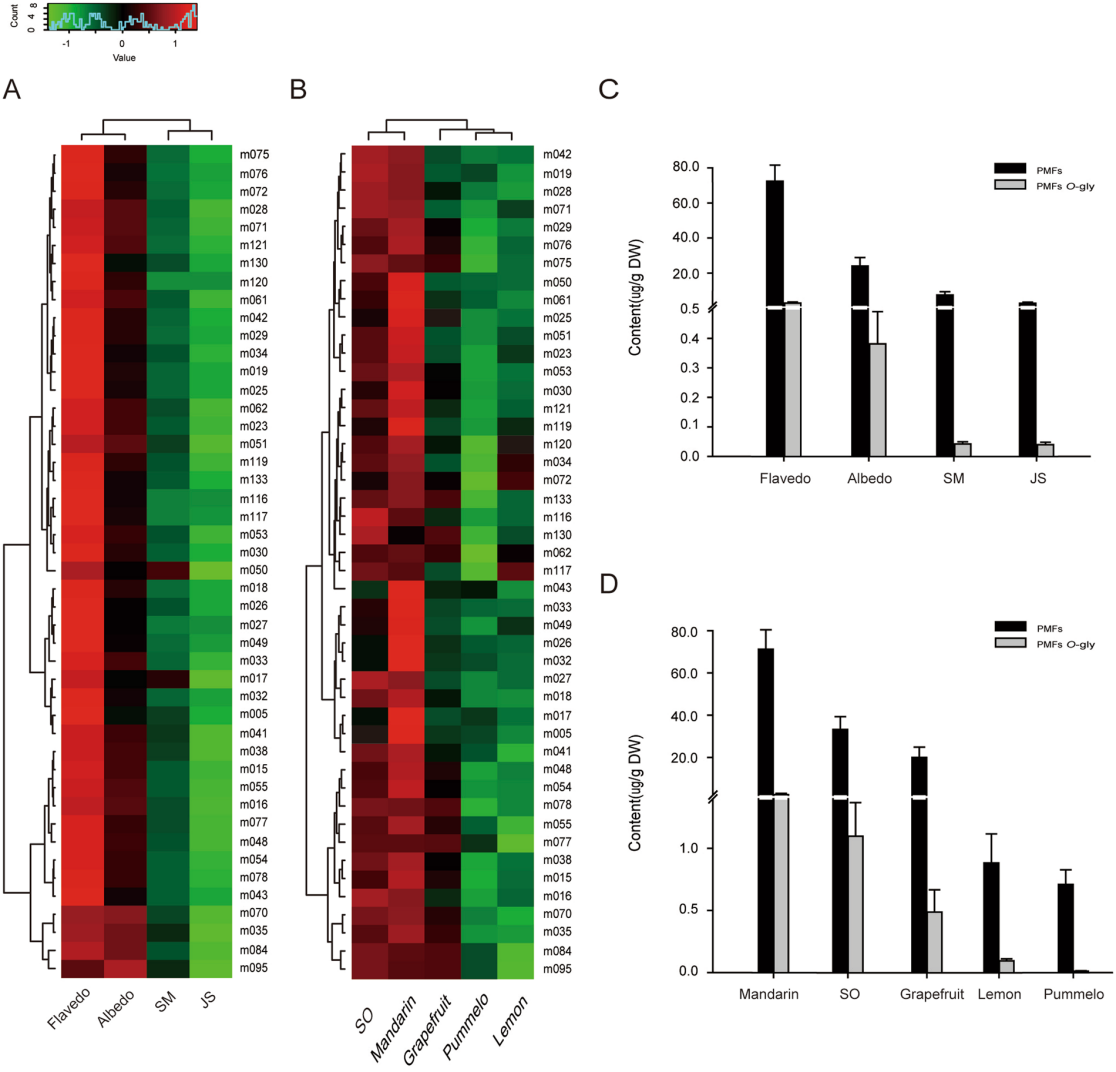
Quantitative analysis of PMFs and *O*-glycosylPMFs in different tissues and varieties of citrus. (**A**) and (**B**) Hierarchically clustered heat map of PMFs and *O*-glycosylPMFs levels of four fruit tissues and five species. The raw data was normalized for clustering analysis. Citrus tissue/species and metabolites are visualized in each column and row. Red and green represent high abundance and low abundance, respectively. (Color key scale above heat map). (**C**) and (**D**) Differential accumulation of PMFs and *O*-glycosylPMFs in various tissues and species (DW, dry weight).

**Figure 5:**
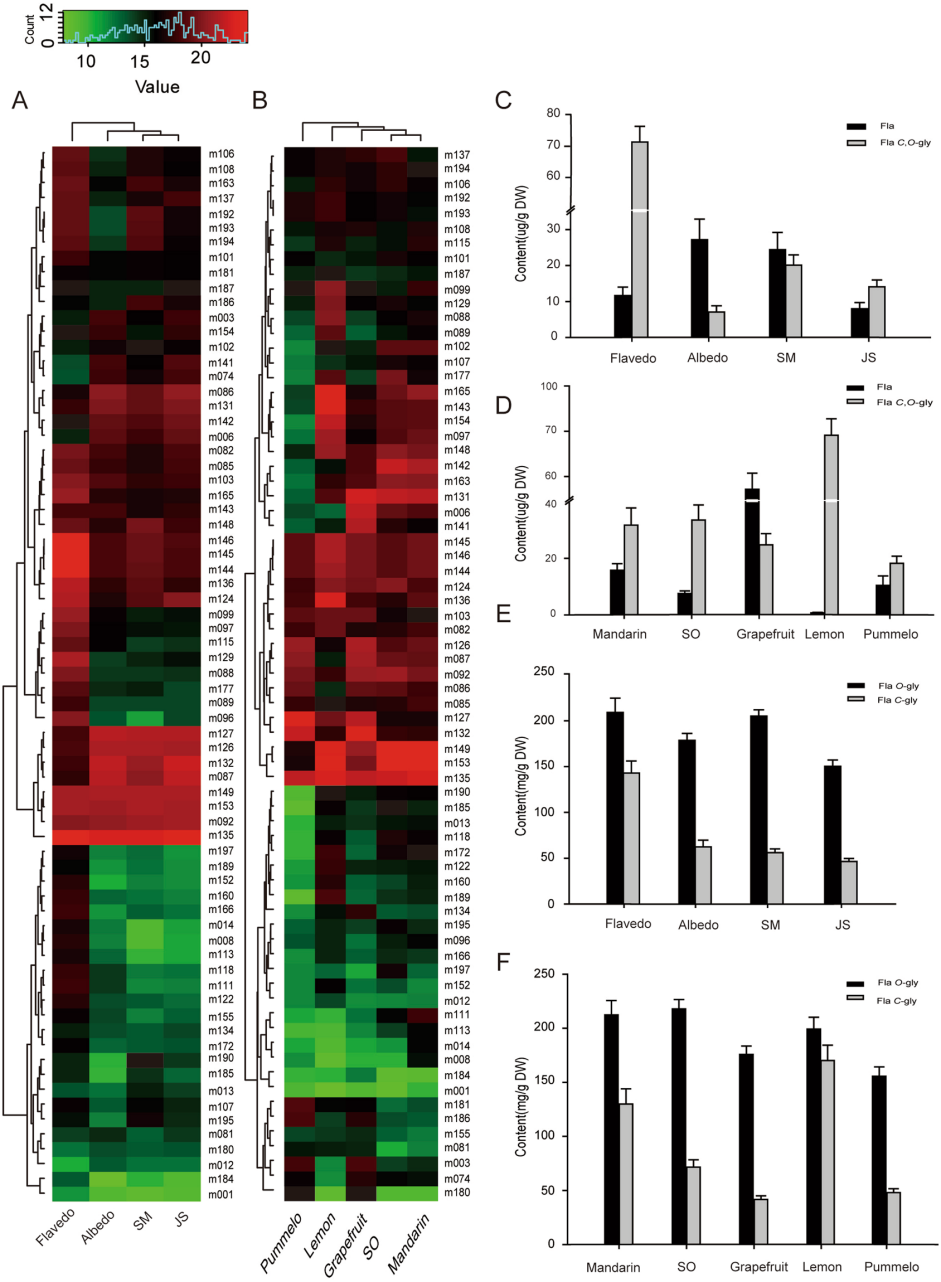
Accumulation patterns of different flavonoids in different fruit tissues and species. (**A**) and (**B**) A heat map for the relative content of the detected flavonoids in citrus. The normalized data was selected for analysis. Rows represent metabolites and column citrus tissue/species. Red represents a high abundance and green a low abundance. (**C**) and (**D**) Contents of flavones and *C*-*O*-glycosylflavonoids in four tissues and five species of citrus. (**E**) and (**F**) The histogram showing the accumulation patterns of *O*-glycosylflavonoids and *C*-glycosylflavonoids in various tissues and species. DW, dry weight.

Hierarchical clustering analysis (HCA) on the pattern of flavonoids in the flavedo, albedo, SM and JS displayed substantial variations, and clearly separated flavedo from other tissues (Figs 4A and 5A). PMFs and their derivatives had the same accumulation patterns in SM and JS tissues, and cluster analysis showed that the two tissues were clustered together.

Furthermore, all investigated tissues contained PMFs and their derivatives, including tetramethoxyflavone, pentamethoxyflavone and hexamethoxyflavone. Compared with the other three tissues, the accumulation of PMFs was highest in flavedo, while the total amount of PMFs and their derivatives was the lowest in JS. (Fig. 4A). Collectively, the facts that the contents of PMFs and PMFs derivatives were the highest and metabolic signals were detected in the flavedo suggested that metabolomes are the most complicated in the flavedo. Therefore, it can be speculated that flavedo, the outer layer of the fruit, may accumulate more metabolites with special physiological functions, such as hydroxylated PMFs, which play an important role in potent inhibition of microorganism growth, and antibacterial, antifungal and antiviral activities^5, 33^. Meanwhile, the accumulation of *O*-glycosylpolymethoxylated flavonoids was also observed in similar trends among four tissues, with the highest levels in the flavedo and the lowest levels being observed in the SM and JS (Fig. 4C).

As for different species, the highest levels of PMFs as well as *O*-glycosylpolymethoxylated flavonoids were found in mandarins, followed by sweet oranges, while significantly lower levels were detected in lemons and pummelos (Fig. 4D). These results are consistent with the results of the above HCA analysis (Fig. 4B). Other flavonoid clusters such as Fla *C*-gly, Fla *O*-gly and Fla *C*, *O*-gly could be categorized into two main groups based on their tissue-specific accumulation patterns. In different fruit tissues, the flavonoids in-group I were mostly detected at higher levels, including a number of glycosylated metabolites (quercetin 3-*O*-rutinoside, luteolin 7-*O*-glucoside and apigenin di-*C*-hexoside); while group II included flavonoid aglycones at lower levels in all fruit tissues, such as naringenin, apigenin and quercetin (Fig. 5A).

Notably, consistent with previous reports^32^, *O*-glycosylated flavonoids were the most abundant flavonoids in all fruit tissues (Fig. 5C and E). In addition, as another evidence for tissue-specific accumulation of flavonoids, *C*-glycosylated flavonoids were preferentially accumulated in the flavedo, followed by the albedo, SM and JS (Fig. 5E).

Furthermore, HCA analysis also revealed the species-specific profiles of flavonoids (Fig. 5B). Lemon had the highest levels of *C*-*O*-glycosylflavonoids while flavonoid aglycones were the most abundant in grapefruits (Fig. 5D). Further analysis revealed that *O*-glycosylated flavonoids were detected at higher levels than *C*-glycosylated flavonoids in all investigated species, and the latter were less accumulated in grapefruits and pummelos (Fig. 5F).

Interestingly, as shown in Fig. 5C, *C*-*O*-glycosylflavonoids were detected at lower levels compared with flavonoid aglycones in all fruit tissues except for the flavedo, indicating that the *C*-*O*-glycosylic process of flavonoids is highly activated in the flavedo. However, in the mixed samples of various tissues (Fig. 5D), *C*-*O*-glycosylflavonoids were detected at higher levels compared with flavonoid aglycones in all investigated *Citrus* species (esp. lemons) except for grapefruits, indicating not only the large contribution of the flavedo-generated flavonoids to the total flavonoid pool of the whole fruit, but also the significantly different genetic control of flavonoid patterns in different *Citrus* species.

### Metabolic diversity of flavonoids in Citrus species

**Figure 6:**
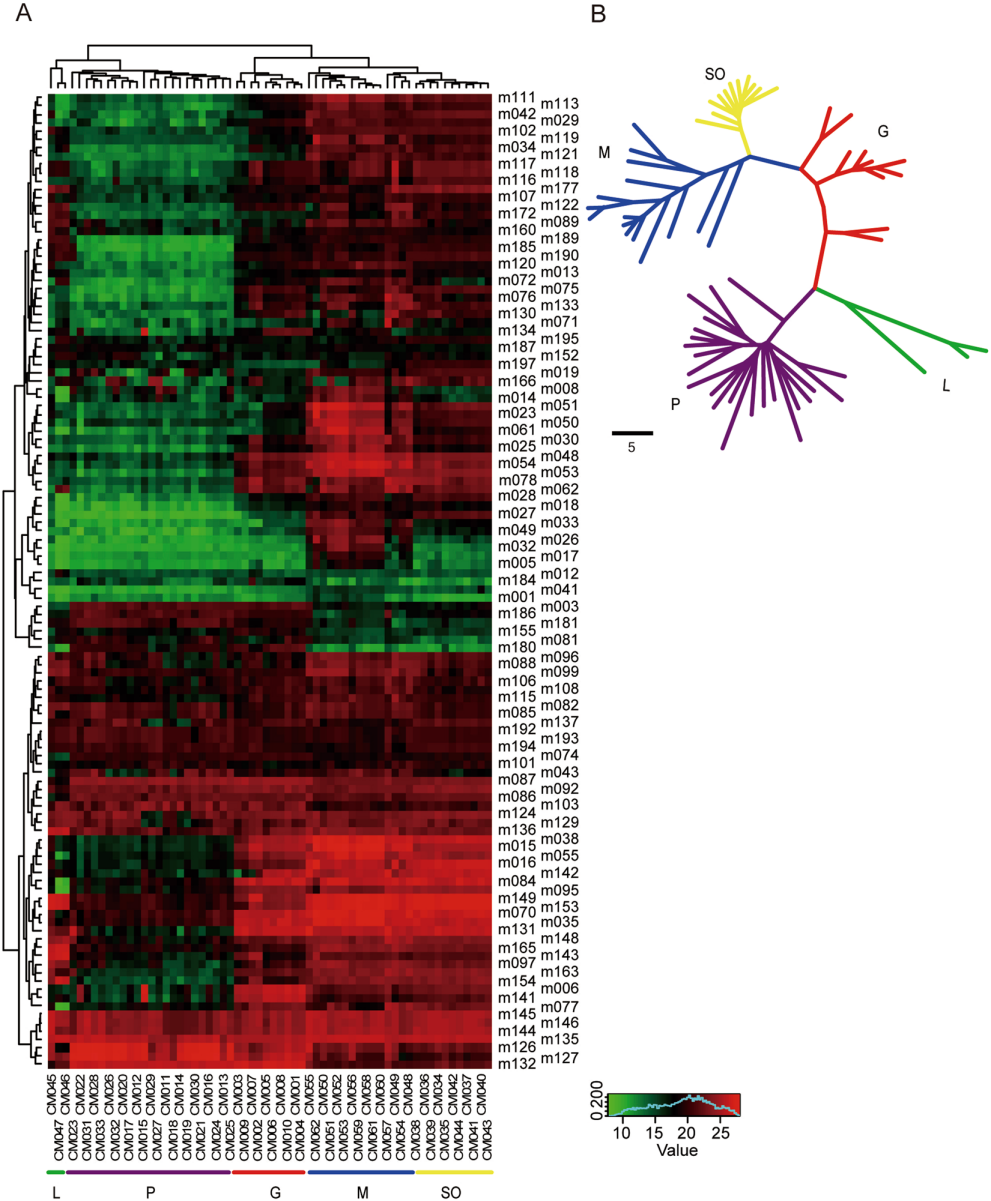
Metabolic diversity and population structure of 62 *Citrus* germplasms based on flavonoid metabolic profiling. (**A**) Heat map of metabolic diversity of flavonoids in 62 *Citrus* germplasms. (**B**) Neighbor-joining tree of 62 *Citrus* germplasms with 117 flavonoids. The five subgroups of the tree are represented by different colors. The scale bar indicates the simple matching distance.

To further study the naturally metabolic diversity of flavonoids in *Citrus* species, mixed samples of four fruit tissues from the 62 germplasms were used for targeted metabolic profiling. Heat map (Fig. 6A) obtained from HCA exhibited the metabolic diversity of flavonoids in *Citrus* species. For example, apigenin *O*-rhamnoside-*O*-rutinoside (m180) was at lower levels in mandarins, sweet oranges and lemons; however, naringenin and luteolin-7-*O*-neohesperidoside-4′-*O*-glucoside (m003 and m186) were at higher levels in pummelos and grapefruits.

Additionally, the results of HCA also showed the great metabolic diversity in the contents of flavonoids among the investigated *Citrus* germplasms (Fig. 6). In Fig. 6, the 62 germplasms were grouped into two clusters, in which lemons and pummelos were separated from other germplasms. Mandarins and sweet oranges, however, were closely clustered, reflecting their relatively close genetic relationship^34^. However, the studies have shown that sweet orange is derived from interspecific hybridization between pummelo (female parent) and mandarin (male parent), and then backcrossed with a male mandarin (sweet orange = (pummelo × mandarin) × mandarin)^35^, while in this study, both HCA results and the neighbor-joining tree (Fig. 6B) demonstrate a closer genetic relationship between Mandarins and sweet oranges.

## Materials and Methods

### Plant materials

62 *Citrus* germplasms used in this study were from a collection of popular/local cultivars in China. These germplasms belong to the species of lemons (*Citrus lemon* [L.] Burm f.), pummelos (*C. grandis* (L.) Osbeck), grapefruits (*C. paradisi* Macf), sweet oranges (*C. sinensis* [L.] Osbeck) and mandarins (*C. reticulata* Marcf.) (Supplementary Table S1), respectively.

Twelve to twenty-one healthy fruits true to its cultivars at commercial maturity were collected randomly from the peripheral canopy of at least three trees and were randomly divided into three biological replicates. The washed material was separated into four tissues, including flavedo, albedo, SM and JS, and immediately placed in liquid nitrogen, and then vacuum freeze-drying using the Heto LyLab 3000 (Heto-Holten A/S, Allerød, Denmark).

### Chemical reagents

Chromatographic-grade acetonitrile, acetic acid and methanol were purchased from Merck (Darmstadt, Germany). The water used as milliQ water was purified using a Millipore purification system (Millipore Corporation). The internal standard lidocaine was purchased from Shanghai New Asiatic Pharmaceuticals Co., Ltd (www.xinyapharm.com/). All standard compounds, including *C*-glycosylflavonoids, flavone, *O*-glycosylflavonoids and polymethoxylated flavonoids (Supplementary Table S3), were purchased from Sigma-Aldrich, USA (http://www.sigmaaldrich.com/united-states.html). All flavonoid standards were dissolved in methanol-dimethyl sulfoxide (50:50, v/v) and stored at −20 °C in darkness.

### Preparation of metabolic samples

The dried material was prepared into a powder using a mixer mill (MM 400, Retsch) under conditions of 1.5 minutes at 30 Hz. Water-soluble metabolites contained in 100 mg powder was extracted with 1.0 ml of the extract (methanol:H_2_O_2_, 50:50, v/v) at 4 °C for 8 hours and then centrifuged at 10, 000 g for 10 min. The supernatant was collected and filtrated (SCAA-104, 0.22 μm pore size; ANPEL), and then analyzed by LC-MS.

To investigate the inter- and intra-species differences in metabolites, mixed samples of four tissues (flavedo, albedo, SM and JS) were analyzed. For each germplasm, 4 ml mixed sample was prepared with 1 ml of the above extracted supernatant from each fruit tissue.

### LC-MS/MS analysis of metabolites

Qualitative metabolic analysis via HPLC-DAD-ESI-QqTOF-MS/MS (6520B, Agilent, USA) was performed in the targeted MS^2^ mode. The UV spectra (DAD) were recorded from 270 to 380 nm. The raw data was analyzed using MassHunter software and the processing method was the same as previously described^32^. Quantitative analysis of metabolites was carried out in the multiple reaction monitoring (MRM) mode by LC-ESI-Q TRAP-MS/MS (4000Q TRAP, ABI, USA). Qualitative and quantitative chromatographic conditions were consistent, and the main parameters were as follows: HPLC: column, shim-pack VP-ODS C18 (pore size 5.0 μm, length 2 × 150 mm); solvent system, water (0.04% acetic acid): acetonitrile (0.04% acetic acid); gradient program, 95:5 V/V at 0 min, 5:95 V/V at 20.0 min, 5:95 V/V at 22.0 min, 95:5 V/V at 22.1 min, 95:5 V/V at 25.0 min; flow rate, 0.25 ml min^−1^; temperature, 40 °C; injection volume: 2 μl^29^. The quantification of flavonoids was performed by calculating the peak area and comparing it to the standard curve drawn by the standard (Supplementary Table S3), including apigenin 8-*C*-glucoside, quercetin 3-*O*-glucoside and 3′,4′,5,6,7,8-hexamethoxyflavone. The standard curve was plotted using the peak area corresponding to four different concentrations of flavonoid standards.

### Statistical analysis

The PCA diagram was drawn using the Mass Profiler Professional (MPP, B.02.01, Agilent), and the analysis parameters were the same as previously described^32^. HCA was performed using R software to study the accumulation patterns and metabolic diversities of metabolites.

The metabolite data containing the 117 relative intensities of metabolites from 62 *Citrus* germplasms. The phylogenetic tree was built from the perspective of metabolomics using pairwise population distance by PHYLIP (version 3.69), and its visualization was performed using TreeView and MEGA5.

## Electronic supplementary material

Supplementary Information

Supplementary Table S1

Supplementary Table S2

Supplementary Table S3

Supplementary Table S4
