# Supplementary material for: Characterization and Metabolic Diversity of Flavonoids in Citrus Species
Source: Sci Rep. 2017 Sep 5;7:10549. doi: 10.1038/s41598-017-10970-2 (PMC5585201; doi:10.1038/s41598-017-10970-2)
Supplement: Supplementary file 1 — Supplementary Information [file 41598_2017_10970_MOESM1_ESM.pdf]

Characterization and Metabolic Diversity of Flavonoids in Citrus Species

Shouchuang Wang[1], Chenkun Yang[1], Hong Tu[2], Junjie Zhou[1], Xianqing Liu[3], Yunjiang Cheng[2], Jie Luo[1,3], Xiuxin Deng[2], Hongyan Zhang[2*] & Juan Xu[2*]

Emails and institutions addresses:

Shouchuang Wang (shouchuang.wang@webmail.hzau.edu.cn), Chenkun Yang (victoryang@webmail.hzau.edu.cn), Hong Tu (tuhong1989@126.com), Junjie Zhou (sand_zhou@webmail.hzau.edu.cn), Xianqing Liu (liuxq@mail.hzau.edu.cn), Yunjiang Cheng (yjcheng@mail.hzau.edu.cn), Jie Luo (jie.luo@mail.hzau.edu.cn), Xiuxin Deng (xxdeng@mail.hzau.edu.cn), Hongyan Zhang (zhanghy@mail.hzau.edu.cn), Juan Xu (xujuan@mail.hzau.edu.cn).

[1]National Key Laboratory of Crop Genetic Improvement and National Center of Plant Gene Research (Wuhan), Huazhong Agricultural University, Wuhan 430070, China.

[2]Key Laboratory of Horticultural Plant Biology (Ministry of Education), College of Horticulture and Forestry Sciences, Huazhong Agricultural University, Wuhan 430070, China.

[3]College of Life Science and Technology, Huazhong Agricultural University, Wuhan 430070, China.

Correspondence and requests for materials should be addressed to H.Z and J.X. (zhanghy@mail.hzau.edu.cn; xujuan@mail.hzau.edu.cn)

Telephone number: 86-27-87286965; Fax number: 86-27-87280016.

Supplementary information:

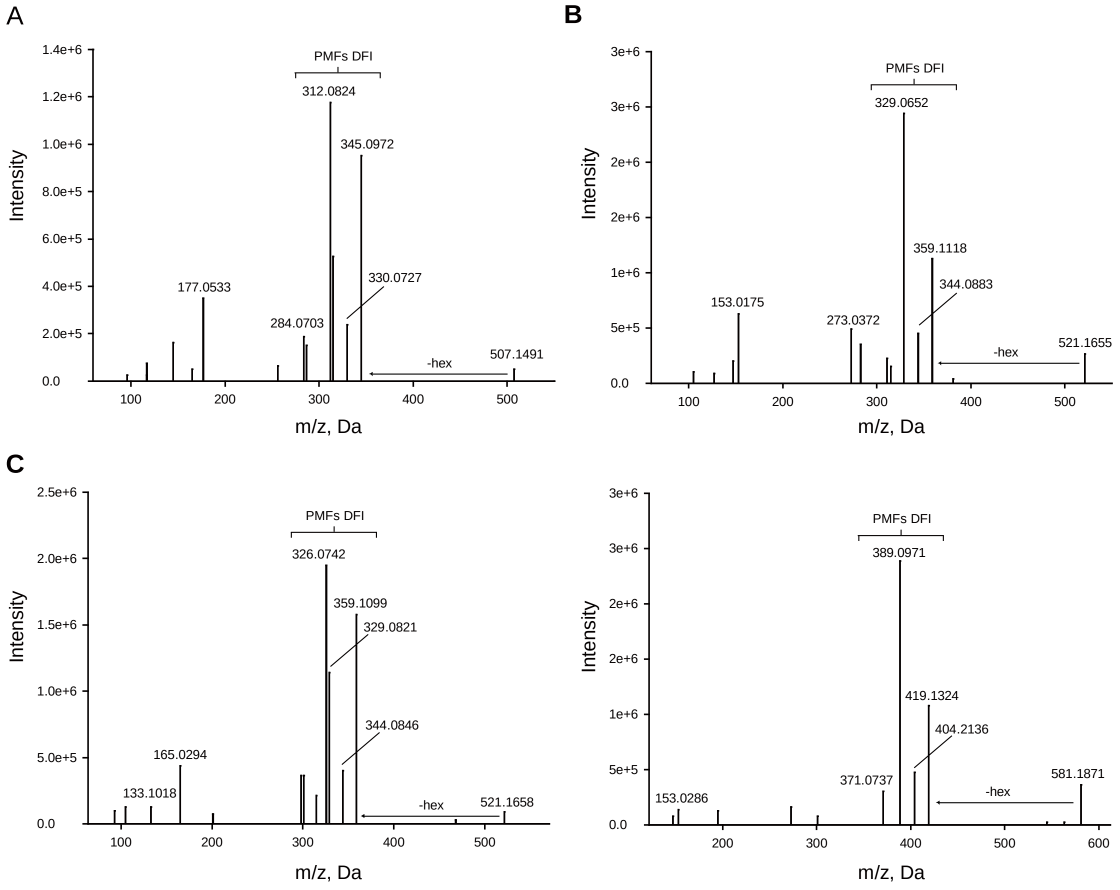

Supplementary Figure 1. Mass spectra and structures of glycosylated PMF in Citrus.

(A) Dihydroxy-trimethoxyflavone-*O*-hexoside (m116). (B) Hydroxy-tetramethoxyflavone-*O*-hexoside (m120). (C) Hydroxy-tetramethoxyflavone-*O*-hexoside (m121). (D) Monohydroxy-hexamethoxyflavone-*O*- hexoside (m130). PMFs DFI, diagnostic fragment ions of polymethoxylated flavonoids.

Supplementary Table S1.

The information of Citrus accessions used in this study.

This table is in a separate sheet.

Supplementary Table S2.

Loadings (correlation coefficients between the original variables and the principal components) of the three PCs (PC1, PC2 and PC3) for each Citrus species.

This table is in a separate sheet.

Supplementary Table S3.

The (almost) non-redundant MS2T library of Citrus flavonoids.

This table is in a separate sheet.

Supplementary Table S4.
Accumulation of 117 flavonoids of various tissues in different Citrus species.
This table is in a separate sheet.
